# Comparative Analysis of Gut Microbiome Dynamics and Dietary Shifts in Three Pollinator Species During Alfalfa Pollination: Insights from Environmental DNA Metabarcoding

**DOI:** 10.3390/insects17060561

**Published:** 2026-05-28

**Authors:** Wei Zhao, Lina Zheng, Zhaoming Wang, Haoran Yan, Jianli Zhang, Guodong Han

**Affiliations:** 1 College of Grassland Science, Inner Mongolia Agricultural University, Hohhot 010018, China; zhaowei@emails.imau.edu.cn; 2 Technology R&D Center, M-Grass Ecology and Environment (Group) Co., Ltd., Hohhot 010010, China

**Keywords:** gut microbiome, pollinator health, environmental DNA metabarcoding, alfalfa pollination, bee sociality

## Abstract

Pollinator health depends critically on gut microbiome composition, yet species-specific responses to agricultural environments remain unclear. We compared gut microbiome dynamics and dietary shifts in *Osmia excavata*, *Megachile rotundata*, and *Apis cerana* during alfalfa pollination using 16S rRNA sequencing and *rbcL* metabarcoding. Alpha diversity revealed distinct patterns: *O. excavata* showed reduced richness but increased evenness after foraging, *M. rotundata* exhibited reduced diversity, while *A. cerana* remained stable. Beta diversity demonstrated microbiome convergence among species after release, indicating environmental filtering. Dietary analysis revealed polylectic foraging dominated by *Citrullus lanatus*, *Peganum nigellastrum*, and *Zea mays*, with minimal alfalfa consumption. Pseudomonadota and Bacillota predominated, with species-specific signatures: *Gilliamella* in *A. cerana*, *Lactobacillus* in *M. rotundata*, and *Bacillus* in *O. excavata*. These findings demonstrate that microbiome assembly reflects host sociality–environment interactions, with solitary bees showing greater plasticity. This study provides a microbial foundation for pollinator conservation in agricultural systems.

## Simple Summary

This study investigates how three bee species, *Osmia excavata*, *Megachile rotundata*, and *Apis cerana*, adjust their gut microbiomes and dietary preferences during alfalfa pollination. Using environmental DNA metabarcoding, we analyzed intestinal microbial communities and plant DNA from bees collected before and after migratory beekeeping. Results reveal that social bees (*A. cerana*) maintain stable core microbiomes dominated by *Gilliamella*, while solitary bees show greater plasticity, with *M. rotundata* exhibiting *Lactobacillus* enrichment and *O. excavata* displaying *Bacillus* prevalence. Dietary analysis indicates polylectic foraging behavior across all species, with minimal direct alfalfa consumption. After-foraging microbiome convergence suggests that environmental factors strongly shape community assembly. These findings provide novel insights into pollinator–microbe interactions in agricultural systems and inform conservation strategies for sustaining pollinator health in managed ecosystems.

## 1. Introduction

Pollinators serve as keystone species in both natural and agricultural ecosystems, facilitating the reproduction of over 87% of flowering plants and contributing to approximately 35% of global food production [1]. Pollinating insects not only directly enhance crop yield and quality but also indirectly maintain ecosystem balance by influencing plant reproduction and evolution [2]. However, in recent years, due to the combined effects of habitat destruction, widespread pesticide use, and climate change, the population size and diversity of pollinating insects have declined sharply, posing a severe challenge to global food security and ecological balance [3]. Alfalfa, as an important leguminous forage crop, highly depends on pollinating insects for its pollination process [4]. Understanding how different pollinator species respond to alfalfa pollination opportunities is therefore essential for developing sustainable agricultural practices that support both crop production and pollinator conservation [5]. Recent evidence suggests that pollinator health and foraging efficiency are intimately linked to their associated microbial communities, which may mediate host responses to environmental stressors and dietary shifts encountered during crop pollination [6,7].

The gut microbiome of insects constitutes a complex microbial ecosystem that profoundly influences host physiology, health, and fitness through multiple mechanistic pathways [8,9,10]. In pollinating insects, studies have demonstrated that the gut microbiota plays vital ecological roles by facilitating the enhanced utilization of food resources such as pollen and nectar, thereby improving nutrient absorption efficiency to sustain normal physiological functions and reproductive capacity [11]. In honeybees, core gut symbionts such as *Gilliamella* and *Bifidobacterium* possess extensive repertoires of carbohydrate-active enzymes that ferment pollen-derived substrates into short-chain fatty acids, which are subsequently utilized by the host for energy metabolism and growth [12]. Honeybees (*Apis mellifera* and *Apis cerana*) have emerged as model systems for understanding host-microbe interactions in social insects. Numerous studies have shown that the bee gut microbiota has a relatively stable community composition, mainly including lactic acid bacteria, bifidobacteria, etc. [7]. These core microorganisms play important roles in bee nutrient absorption and immune defense. For example, lactic acid bacteria can produce organic acids, regulate the acid–base balance of the bee gut, and inhibit the growth of harmful bacteria [13]. At the same time, the gut microbiota community structure of bees is also affected by many factors, such as environmental factors, diet structure, and the developmental stage of bees [14]. However, most of the current research on bee gut microbiota community structure focuses on a single species or specific environments, and there is a lack of systematic research on the dynamic changes in gut microbiota community structure.

A fundamental dichotomy exists in microbiome assembly patterns between social and solitary bees, reflecting divergent transmission pathways and ecological selection pressures [15,16]. Social bees, including honeybees and bumblebees, have relatively stable microbiota, which are mainly acquired through social transmission [17]. This social mode of transmission promotes microbiome stability across generations and geographical regions, resulting in co-evolved symbioses where specific bacterial lineages have co-diversified with their hosts over evolutionary timescales [18,19]. In contrast, solitary bees, which comprise the vast majority of bee species, lack social transmission mechanisms and must acquire their gut microbiota from environmental sources, including floral nectar, pollen, nesting materials, and soil [20]. Studies on *Osmia* species reveal that solitary bee microbiomes exhibit high inter-individual variability and are dominated by environmentally acquired bacteria, including plant-associated and soil-derived taxa [21,22]. The gut bacterial communities of *Osmia excavata* across developmental stages demonstrate that larvae acquire microbes primarily from pollen provisions, with community composition shifting dramatically during metamorphosis and following emergence from cocoons [23]. Notably, solitary bee microbiomes show greater plasticity in response to local environmental conditions, with urbanization, floral resource availability, and landscape composition significantly shaping community structure [24]. This difference may lead to different adaptabilities and resistances of social and solitary bees in response to environmental changes and pathogen infections.

Environmental DNA metabarcoding has revolutionized the study of host-associated microbial communities and trophic interactions, with great potential and significant advantages, offering unprecedented resolution in characterizing both gut microbiomes and dietary composition from single samples [18,22]. This technology can quickly and accurately identify the microbial species and diet composition in samples by high-throughput sequencing of DNA in environmental samples [25]. Compared with traditional methods, environmental DNA metabarcoding technology has higher sensitivity and resolution, and can detect low-abundance microorganisms and food components [26]. In addition, this technology is non-invasive and does not cause harm to the research objects, making it suitable for the study of wild pollinating insects [22]. The ability to link dietary composition directly with gut microbial community structure represents a particular advantage for understanding how foraging behavior shapes microbiome assembly, especially in solitary bees where environmental acquisition predominates [27]. As sequencing costs continue to decrease and bioinformatic pipelines become increasingly accessible, environmental DNA metabarcoding is poised to become the standard approach for integrative studies of pollinator ecology and microbiology [28].

Despite growing recognition of the gut microbiome’s importance for pollinator health, critical knowledge gaps remain regarding how different bee species respond to shared environmental contexts and resource opportunities [29]. Agricultural landscapes, particularly crop pollination events, provide ideal natural experiments for such comparative analyses, as multiple pollinator species converge on the same floral resources within a defined spatial and temporal window [30]. However, few studies have simultaneously tracked microbiome dynamics and dietary composition across multiple bee species during active crop pollination, limiting our understanding of how foraging behavior and sociality interact to shape gut community assembly [31]. The present study addresses these knowledge gaps by conducting a comparative analysis of three bee species, *Osmia excavata* (mason bees), *Megachile rotundata* (leafcutter bees), and *Apis cerana* (Asian honeybee), during alfalfa pollination in a shared agricultural system. By integrating 16S rRNA amplicon sequencing for gut microbiome characterization with plant DNA metabarcoding for dietary analysis, we aim to elucidate how pollination behavior shapes microbial community dynamics and whether dietary differentiation among species drives microbiome divergence. This integrative approach will provide insights into the ecological processes governing pollinator–microbe interactions in agricultural contexts and inform conservation strategies for maintaining pollinator health in managed ecosystems.

## 2. Materials and Methods

### 2.1. Research Design and Sample Collection

#### 2.1.1. Experimental Design and Grouping

This study selected three types of bees as the research subjects, namely *Osmia excavata* (BF), *Megachile rotundata* (QYF), and *Apis cerana* (ZHF). Two sampling time points were set based on the timing of bee release, namely before migratory beekeeping (Q) and after migratory beekeeping (H). The aim was to compare changes in the gut microbial community structure of different bee species before and after release and to characterize the detected dietary plant composition of the three bee species after release. Three biological replicates were established for each species at each time point, with each replicate consisting of intestinal contents pooled from 15 individuals, to ensure data reliability and the validity of statistical analysis. All pollinator colonies were deployed in the alfalfa experimental field in Urat Middle Banner, Bayannur City, Inner Mongolia Autonomous Region, China. Based on the alfalfa flowering period, the bee release date was 10 June 2025, and the bee recovery date was 26 August 2025.

Floral resource abundance in and around the alfalfa field was not quantitatively surveyed during the sampling period; therefore, dietary plant DNA profiles were interpreted as detected dietary composition rather than direct evidence of floral preference or avoidance. Because plant dietary metabarcoding was conducted only for after-release samples, the results do not allow direct comparison of before-release and after-release dietary shifts.

#### 2.1.2. Sample Collection and Preservation

The bee samples were processed immediately after being collected at the corresponding time points. All samples were handled under sterile conditions to minimize the possibility of contamination by exogenous microorganisms. Bee individuals were first immobilized and euthanized by freezing at −20 °C before dissection. Whole guts were dissected under sterile conditions using sterilized forceps and dissecting needles. The dissected gut tissues were rinsed with sterile phosphate-buffered saline (PBS), transferred into sterile centrifuge tubes, and immediately frozen in liquid nitrogen. All samples were sent to Magigene One Health Biotechnology Co., Ltd., Suzhou, China for DNA extraction, amplification, and sequencing.

### 2.2. DNA Extraction, Amplification and Sequencing

#### 2.2.1. Gut Microbiota: Extraction, Amplification, and Sequencing of 16S rRNA Gene

After extracting genomic DNA using the MOBIO Power Soil^®^ DNA Isolation Kit (MO BIO Laboratories, Inc., Carlsbad, CA, USA) according to the instructions, 1% agarose gel electrophoresis was used to check the integrity and purity of the DNA, and Nano Drop One (Thermo Fisher Scientific Inc., Wilmington, DE, USA) was used to detect the concentration and purity of the DNA. PCR amplification and product electrophoresis were performed using the 16S rRNA gene as the template. According to the selection of the sequencing region, primers with barcodes and Premix Taq (Takara Bio, Shiga, Japan) were used for PCR amplification, as shown in Table 1. After comparing the concentrations of the PCR products using Gene Tools Analysis Software (Version 4.03.05.0, Syn Gene) (Syngene, Cambridge, UK), the required volume for each sample was calculated based on the equal mass principle, and the PCR products were mixed. The PCR mixture was recovered using the E.Z.N.A.^®^ Gel Extraction Kit (Omega Bio-tek, Inc., Norcross, GA, USA), and the target DNA fragments were eluted with TE buffer.

Library construction was carried out following the standard protocol of the ALFA-SEQ DNA Library Prep Kit, and the size of the library fragments was evaluated on the Qsep400 High-Throughput Nucleic Acid & Protein Analysis System (Hangzhou Houze Biotechnology Co., Ltd., Hangzhou, China). The concentration of the library was measured using a Qubit4.0 (Thermo Fisher Scientific, Waltham, MA, USA). The constructed amplicon libraries were subjected to PE250 sequencing on either the Illumina or MGI platform (Guangdong Magigene Biotechnology Co., Ltd., Guangzhou, China).

#### 2.2.2. Plant DNA: Extraction, Amplification, and Sequencing of Chloroplast rbcL

According to the instructions of the TIANamp Marine Animals DNA Kit (TIANGEN Biotech, Beijing, China), genomic DNA extraction is carried out, and the concentration of DNA is detected using Qubit. PCR primers selected are specific primers targeting the target region of the chloroplast *rbcL* gene for amplification, and they also contain a Barcode sequence. The primer information is shown in Table 1. Based on the principle of Qubit concentration and quality, the volume required for each sample is calculated, and the PCR products of each sample are mixed and gel-cut for recovery. Subsequent library construction follows the standard process of the ALFA-SEQ Amplification Library Prep Kit. The library quality is evaluated using the Qubit and QSEP400 high-throughput nucleic acid protein analysis system. After completion, the library is sequenced on the Illumina NovaSeq 6000 platform. The original image data files obtained from sequencing are analyzed through base calling to convert them into raw sequencing sequences (raw reads). The results are stored in FASTQ (abbreviated as fq) file format, including the sequence information of the sequences and the corresponding sequencing quality information.

### 2.3. Data Analysis Methods

#### 2.3.1. Analysis of Gut Microbial Data

Based on the reads obtained from the second-generation sequencing, the original reads were first quality-controlled using the Fastp software (version 0.14.1), with the adapters removed and low-quality reads filtered out, including: filtering reads with a length less than 200 bp; filtering reads with an average base quality value lower than 15; filtering reads with an unqualified base proportion exceeding 40%.

After obtaining high-quality data, the filtered paired-end reads were then spliced using the USEARCH software (version 10.0.240). Sequence correction was performed using FramBot v 1.0, followed by redundancy removal and primer removal to obtain non-redundant sequences. Finally, UPARSE was used for clustering to obtain representative sequences.

Subsequently, the obtained representative sequences were compared with the database Silva (v119/v123/v128/v132/v138), and species annotation results were obtained. Based on the species annotation of the OTU table, further analysis was conducted.

#### 2.3.2. Analysis of Plant Data

Based on the reads obtained from the second-generation sequencing, the original reads were first quality-controlled using the Fastp software (version 0.12.4), with the adapters removed, and low-quality reads were filtered out, including: filtering reads shorter than 100 bp; filtering reads with more than 5 N bases; filtering reads with an average base quality value lower than 20; and filtering reads with an excessive percentage of unqualified bases (more than 3%). The quality of the reads before and after filtering was evaluated using the FastQC software (version 0.11.9). Then, the paired-end reads after filtering were spliced using the USEARCH software (version 11.0.667) (default selection of 350,000 reads, or all if insufficient), and primer sequences and sequences with sequencing errors exceeding 1% were removed according to the alignment results of the primers at both ends. At the same time, the length of the spliced sequences was maintained within the specified length range of the amplification product. Depending on the need, the human amplimer sequences in the samples were filtered by comparing with the human target macro barcode sequence.

After filtering, all the spliced sequences of the samples were merged, and duplicate sequences were removed using the VSEARCH v2.30.0, and representative sequences with an abundance greater than 4 were retained. Then, the representative sequences were filtered using the uchime3_denove algorithm of USEARCH software to remove chimeric sequences, and the OTU representative sequences were obtained using the UPARSE algorithm. Finally, the spliced sequences of the merged samples were compared with the OTU sequence set to obtain the final OTU table. The obtained *rbcL* OTU representative sequences were compared with the reference sequences in the target plant barcode database using BLAST (version 2.2.31). Sequences with alignment coverage <90% and sequence identity <80% were removed as low-confidence matches. For the retained sequences, taxonomic assignment was performed using hierarchical sequence-identity thresholds. Specifically, OTUs with sequence identity ≥98%, ≥95%, ≥90%, ≥85%, and ≥80% were assigned to the species, genus, family, order, and class levels, respectively.

#### 2.3.3. Software and Data Visualization

All data were stored and preliminarily processed using Microsoft Excel 2020. Statistical analyses and data visualization were performed in R version 4.5.2 using the packages vegan, phyloseq, ggplot2, dplyr, tidyr, ggrepel, RColorBrewer, and patchwork. Additional figures were generated using Origin 2025, and final figure layout and optimization were performed using Adobe Illustrator 2023.

## 3. Results

### 3.1. Sequencing Data Results and Quality Control

After high-throughput sequencing, quality control, removal of chimeras and haplotypes, and OTU clustering, a total of 953,081 sequences and 519 OTUs were obtained from 18 samples of the intestinal microbiota, and 931,203 sequences and 31 OTUs were generated from 9 samples for dietary analysis (Tables S1 and S2). Based on a sequence similarity of 97%, all sequences were subjected to homology comparison to obtain OTUs, and the species annotation results were statistically analyzed to obtain 2 kingdoms (Bacteria, Archaea), 17 phyla, 30 classes, 67 orders, 100 families, and 148 genera, representing 163 species. Archaeal reads assigned to Nitrososphaeraceae were detected only in two samples, BFQ3 and QYFQ3, and were not consistently observed across biological replicates. Given their sporadic occurrence, these reads were treated cautiously as rare signals or potential contaminants.

**Figure 1:**
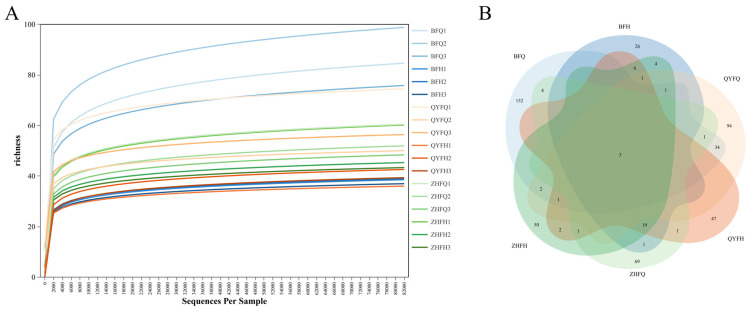
Rarefaction curves and OTU-sharing patterns of gut microbiota across sample groups. (**A**) Rarefaction curves of gut microbial OTUs in different samples. (**B**) Venn diagram showing shared and unique OTUs among the six treatment groups (BFQ, BFH, QYFQ, QYFH, ZHFQ, and ZHFH). Numbers in overlapping regions indicate shared OTUs, and numbers in non-overlapping regions indicate unique OTUs.

The dilution curve results showed that each sample generally tended to be flat, indicating that the sequencing data volume of the samples was reasonable (Figure 1A). Among the intestinal microbiota, BFQ2 had the largest number of OTUs (95), while QYFH1 had the fewest (34); the top three sequences in terms of quantity were BFQ1, BFQ2, and BFQ3, with 82,661, 82,060, and 80,315 sequences respectively; the least were QYFQ1, QYFQ2, and QYFQ3, with 7780, 3144, and 1836 sequences respectively. In the dietary analysis after bee release, the number of OTUs was concentrated between 28 and 30; the sequence quantity was the highest for BFH2, with 170,660 sequences; the lowest was BFH3, with only 30,408 sequences.

The six-element Venn diagram shows the shared OTU situations among different samples (Figure 1B). All samples have 3 common OTUs; BFQ has the largest number of unique OTUs (152), while BFH has the smallest number of unique OTUs (26).

### 3.2. Composition Characteristics of the Intestinal Microbial Community

**Figure 2:**
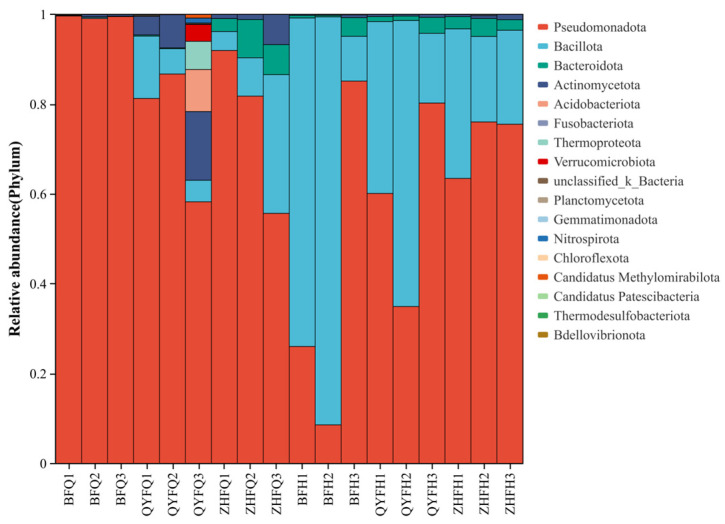
Relative abundance of gut microbial taxa at the phylum level across all samples. Different colors represent different microbial phyla.

At the phylum level (Figure 2), the top five species were *Pseudomonadota* (70.38%), *Bacillota* (26.37%), *Bacteroidota* (2.28%), *Actinomycetota* (0.89%), and *Acidobacteriota* (0.02%). Apart from *Bacillota*, which are the dominant bacterial phyla in BFH1, BFH2, and QYFH2 with relative abundances of 26.05%, 8.60%, and 34.95% respectively, the dominant bacterial phyla of most samples are *Pseudomonadota*.

At the genus level, the dominant annotated genera included *Gilliamella* (32.58%), *Lactobacillus* (19.86%), *Bacillus* (6.03%), and *Snodgrassella* (5.56%). Sequences assigned to *Enterobacterales* but not resolved to the genus level accounted for 26.30% and were reported as unclassified *Enterobacterales*. Apart from *Bacillota*, which are the dominant bacterial phyla in BFH1, BFH2, and QYFH2 with relative abundances of 26.05%, 8.60%, and 34.95% respectively, the dominant bacterial phyla of most samples are *Pseudomonadota*.

Taxon-level statistical analysis showed that none of the dominant bacterial phyla differed significantly among the six species–time groups after FDR correction. Although *Bacteroidota*, *Actinomycetota*, and *Bacillota* showed nominal differences among the six groups before correction, these differences did not remain significant after FDR correction (minimum FDR-adjusted *p* = 0.066). When samples were pooled by sampling time, *Bacillota* differed significantly between before- and after-release samples (χ^2^ = 9.827, df = 1, FDR-adjusted *p* = 0.010), whereas *Pseudomonadota* showed only a nominal difference before correction (χ^2^ = 5.476, df = 1, *p* = 0.019; FDR-adjusted *p* = 0.058). No dominant bacterial phylum differed significantly among bee species when the before- and after-release periods were analyzed separately.

At the genus level, 14 dominant genera or genus-level taxonomic groups differed significantly among the six species–time groups after FDR correction. These included *Bifidobacterium*, *Bombella*, unclassified *Orbaceae*, unclassified *Enterobacteriaceae*, unclassified *Comamonadaceae*, *Snodgrassella*, *Apibacter*, unclassified *Micrococcaceae*, *Gilliamella, Lactobacillus*, unclassified *Yersiniaceae*, the *Methylobacterium*-*Methylorubrum* group, *Bacillus*, and *Sphingomonas* (Kruskal–Wallis tests, FDR-adjusted *p* < 0.05). When sampling periods were analyzed separately, interspecific differences before release were detected for multiple dominant genus-level taxa, including *Apibacter, Bifidobacterium*, *Bombella*, *Gilliamella*, *Snodgrassella*, unclassified *Enterobacteriaceae*, unclassified *Orbaceae*, the *Methylobacterium*-*Methylorubrum* group, unclassified *Micrococcaceae*, *Sphingomonas*, unclassified *Comamonadaceae*, unclassified *Enterobacterales*, and unclassified *Yersiniaceae* (FDR-adjusted *p* < 0.05). In contrast, no dominant genus-level taxon remained significantly different among the released bee species after FDR correction. These results indicate that genus-level differences among bee species were more evident before release and became less statistically distinguishable after release.

### 3.3. Analysis of Intestinal Microbial Alpha Diversity

**Figure 3:**
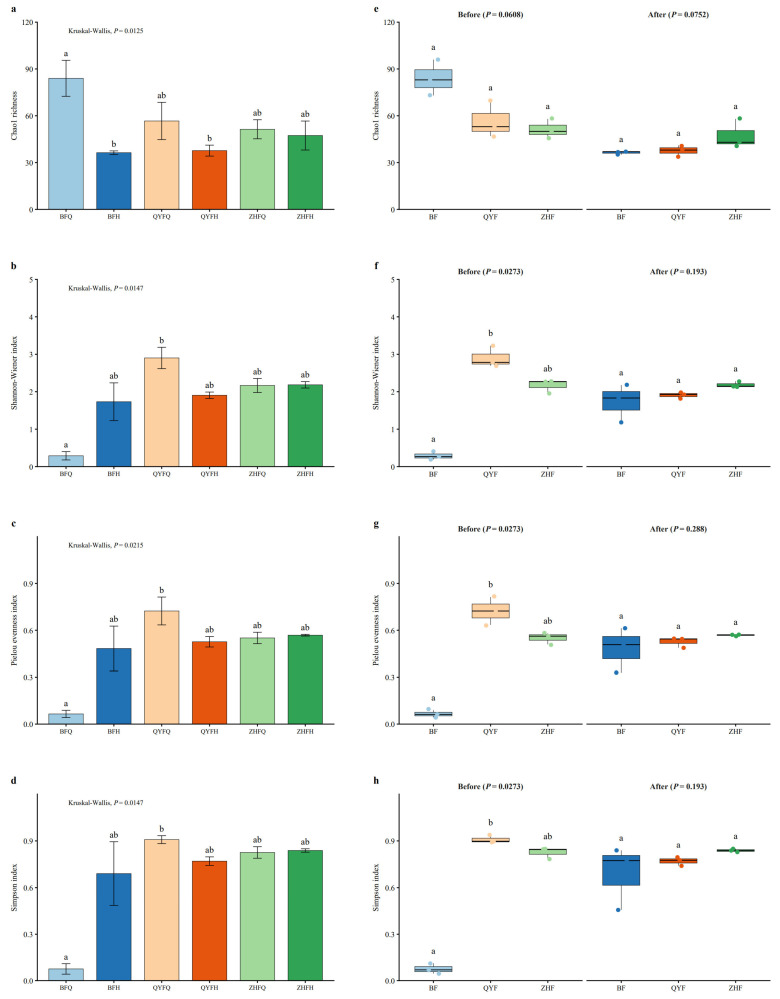
Alpha diversity analysis of gut microbial communities. (**a**–**d**) Mean ± SD values of Chao1 richness, Shannon–Wiener diversity, Pielou’s evenness, and Simpson diversity among the six species–time groups. Kruskal–Wallis tests were used to compare differences among the six groups, followed by Dunn’s post hoc tests. Different letters indicate significant differences among groups (adjusted *p* < 0.05). (**e**–**h**) Boxplots showing Chao1 richness, Shannon–Wiener diversity, Pielou’s evenness, and Simpson diversity before and after release. Kruskal–Wallis tests were used to compare differences among bee species within each sampling period, followed by Dunn’s post hoc tests where appropriate. For panels (**e**–**h**), letters indicate Dunn’s post hoc comparisons among bee species within each sampling period; groups sharing the same letter are not significantly different, whereas groups with different letters differ significantly (adjusted *p* < 0.05). Points represent biological replicates.

Alpha diversity analysis showed that gut microbial diversity changed differently among bee species before and after migratory beekeeping (Figure 3, Table S3). In the BF group, Chao1 richness decreased after release, whereas Shannon diversity, Pielou’s evenness, and Simpson diversity increased, indicating reduced richness but increased evenness and overall diversity. In the QYF group, all four alpha diversity indices decreased after release. In contrast, the ZHF group showed comparatively small changes across the four indices. At the individual-sample level, QYFQ3 had the highest Shannon diversity, Pielou’s evenness, and Simpson diversity values, at 3.227, 0.813, and 0.937, respectively, whereas BFQ1 had the lowest corresponding values, at 0.191, 0.043, and 0.045, respectively. The highest and lowest Chao1 richness values were observed in BFQ2 (96) and QYFH1 (34), respectively.

Statistical analyses supported significant differences in alpha diversity among the six species–time groups. Kruskal–Wallis tests showed significant differences for Chao1 richness (χ^2^ = 14.548, df = 5, *p* = 0.0125), Shannon diversity (χ^2^ = 14.146, *p* = 0.0147), Pielou’s evenness (χ^2^ = 13.211, *p* = 0.0215), and Simpson diversity (χ^2^ = 14.146, *p* = 0.0147). Dunn’s post hoc comparisons are indicated by letter annotations in Figure 3.

When the two sampling periods were analyzed separately, interspecific differences were more evident before release than after release. Before release, Shannon diversity, Pielou’s evenness, and Simpson diversity differed significantly among bee species (Kruskal–Wallis tests, *p* = 0.0273 for all three indices), whereas Chao1 richness showed a marginal but non-significant difference (*p* = 0.0608). After release, none of the four alpha diversity indices differed significantly among bee species (Chao1 richness, *p* = 0.0752; Shannon diversity, *p* = 0.193; Pielou’s evenness, *p* = 0.288; Simpson diversity, *p* = 0.193).

Aligned rank transform ANOVA was used to test the interaction between species and sampling time. Significant interactions were detected for Chao1 richness (F = 10.792, *p* = 0.00208), Shannon diversity (F = 35.683, *p* < 0.001), Pielou’s evenness (F = 43.965, *p* < 0.001), and Simpson diversity (F = 53.375, *p* < 0.001), indicating that temporal changes in alpha diversity differed among bee species. Specifically, BF showed decreased richness but increased evenness and diversity after release, QYF showed decreases across all four indices, and ZHF showed comparatively limited changes.

### 3.4. The β Diversity Analysis Revealed the Differences in the Intestinal Microbial Community Structures Among Different Bee Species

**Figure 4:**
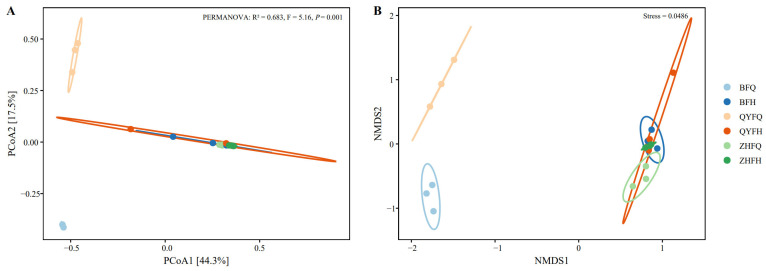
Beta diversity analysis of gut microbial communities. (**A**) Principal coordinates analysis (PCoA) based on the Bray–Curtis distance matrix. (**B**) Non-metric multidimensional scaling (NMDS) based on the Canberra distance matrix. Different colors represent the six sample groups: BFQ, BFH, QYFQ, QYFH, ZHFQ, and ZHFH. Circles indicate individual samples, and colored ellipses indicate the 95% confidence regions of the corresponding groups. The percentages shown on the PCoA axes indicate the proportion of variation explained by each axis. The stress value in the NMDS plot indicates the goodness of fit of the ordination. PERMANOVA based on the Bray–Curtis distance matrix was used to test differences in gut microbial community composition among the six groups.

Beta diversity analysis showed differences in gut microbial community structure among the six sample groups (Figure 4). In the PCoA based on the Bray–Curtis distance matrix, the first two principal coordinates explained 44.3% and 17.5% of the total variation, respectively. The six groups showed different degrees of separation in the ordination space, indicating variation in community composition among the sample groups. Compared with the before-release samples, several after-release samples were positioned more closely in the ordination space, suggesting a tendency toward reduced between-group variation after migratory beekeeping.

The NMDS analysis based on the Canberra distance matrix showed a broadly similar distribution pattern to the PCoA result. The low stress value of 0.0486 indicated that the NMDS ordination provided a reliable representation of the community dissimilarities. To statistically verify the differences observed in the PCoA, PERMANOVA was performed based on the Bray–Curtis distance matrix. The result confirmed significant differences in gut microbial community composition among the six groups (R^2^ = 0.683, F = 5.160, *p* = 0.001). These results suggest that gut microbial community structure differed among sample groups before and after migratory beekeeping.

### 3.5. Analysis of the Dietary Composition of Bees Based on the rbcL Macrobarcode

**Figure 5:**
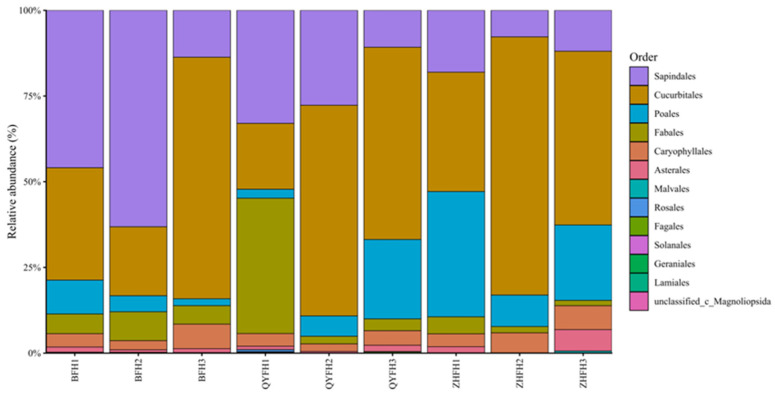
Relative abundance of dietary plant taxa at the order level in after-release bee samples based on *rbcL* metabarcoding. Different colors represent different plant orders. BFH, QYFH, and ZHFH represent after-release samples of the three bee species.

The 931,203 sequences were clustered into 31 OTUs. After species information annotation of the OTUs, they all belonged to Viridiplantae, Streptophyta, Magnoliopsida, and were respectively classified into 13 Orders, 16 Families, and 27 genera of 29 Species (Figure 5, Table S2).

The distribution of plant DNA profiles differed among after-release samples, suggesting variation in the detected dietary plant composition among bee individuals and species. Among them, Sapindales (only *Peganum nigellastrum*) and Cucurbitales (including *Citrullus lanatus*, *Cucurbita pepo*, and *Cayaponia* sp.) occupy a relatively dominant position in all samples, and Sapindales has the most dominant position in BFH2, while Cucurbitales only accounts for a relatively small proportion in BFH2 and QYFH1. Poales appears more frequently in the three samples of ZHFH, but has a lower dominance in other samples. In QYFH1, Fabaceae accounted for a considerable proportion, mainly because *Medicago sativa* contributed 16,398 sequence reads in this sample.

Across the after-release samples, the most abundant plant taxa were *Citrullus lanatus*, *Peganum nigellastrum*, *Zea mays*, *Medicago sativa*, and *Axyris hybrida*. Among them, *C. lanatus* and *Z. mays* are important crops, *M. sativa* is a forage crop, whereas *P. nigellastrum* and *A. hybrida* are non-crop plants.

At the species-level assignment, a substantial proportion of plant reads remained unclassified, with an average relative abundance of 50.0% across after-release samples. Among the classified plant taxa, the dominant detected taxa were *Citrullus lanatus*, *Peganum nigellastrum*, *Zea mays*, *Medicago sativa*, and *Axyris hybrida*, with mean relative abundances of 22.8%, 12.9%, 6.4%, 3.7%, and 2.1%, respectively.

To evaluate whether detected dietary plant DNA composition differed among bee species after release, PERMANOVA was performed based on Bray–Curtis dissimilarities. The result showed no significant difference among the three bee species (R^2^ = 0.182, F = 0.667, *p* = 0.641). Exploratory Kruskal–Wallis tests with Benjamini–Hochberg FDR correction were further conducted for dominant plant taxa. No dominant plant taxon remained significant after FDR correction (all FDR-adjusted *p* ≥ 0.504).

## 4. Discussion

Our study provides the first systematic comparison of gut microbiome dynamics and dietary shifts in three pollinator species, *Osmia excavata* (mason bee), *Megachile rotundata* (leafcutter bee) and *Apis cerana* (Asian honeybee), during active alfalfa pollination. The dietary analysis revealed distinct foraging patterns among these species. Alfalfa (*Medicago sativa*) pollen constituted a relatively minor proportion of the overall diet across all three bee species, with *Citrullus lanatus* (watermelon), *Peganum nigellastrum*, and *Zea mays* (maize) dominating the pollen profiles. This finding aligns with recent studies demonstrating that *M. rotundata*, despite being commercially managed primarily for alfalfa pollination, exhibits polylectic foraging behavior and readily utilizes alternative floral resources when available [4,5]. The capacity to exploit diverse pollen sources is ecologically significant, as polyfloral diets have been shown to enhance bee health by providing balanced nutritional profiles and reducing susceptibility to pathogens [32,33]. The dietary diversity observed in our study supports the notion that a varied diet can improve bee health and resilience, as highlighted by [7], who emphasized the role of a diverse microbiome in mediating host responses to dietary shifts.

The gut microbiome analysis revealed both shared and species-specific patterns across the three pollinators. All species exhibited dominance of *Pseudomonadota* and *Bacillota*, consistent with previous reports on bee gut microbiomes [29]. However, distinct functional signatures emerged: *A. cerana* and *O. excavata* showed enrichment of *Gilliamella* and *Lactobacillus*, respectively, while *M. rotundata* displayed a unique microbial profile characterized by reduced alpha diversity but stable community structure. The after-foraging convergence of microbiome composition toward *Lactobacillus* dominance in *M. rotundata* is particularly intriguing, as this genus has been implicated in pollen digestion, immune modulation, and pathogen defense in solitary bees [23,27]. The high abundance of *Enterobacterales* in before-foraging *O. excavata* samples suggests environmental acquisition from nesting materials, whereas the shift toward *Gilliamella* and *Snodgrassella* in *A. cerana* reflects the characteristic core microbiome of social bees maintained through vertical transmission [12,29]. In contrast, *Bacillus* sp. was prevalent in the mason bee (*Osmia excavata*), suggesting a potential role in nitrogen fixation or pathogen suppression, as suggested by recent studies on solitary bee microbiomes [21]. These findings support the emerging view that while social bees harbor conserved, host-specific microbiomes, solitary bees exhibit greater plasticity in response to environmental microbial availability [20].

The ecological and agricultural significance of *M. rotundata* extends beyond its role as an efficient alfalfa pollinator. As the second most widely managed pollinator globally, this species contributes to seed yield increases of 50–130% in alfalfa fields [34,35]. Our microbiome data suggest that the stable *Lactobacillus*-dominated community in *M. rotundata* may confer adaptive advantages under agricultural intensification. Recent research has demonstrated that *Lactobacillus* spp. enhances host immunity through Toll pathway activation and antimicrobial peptide production, providing protection against pathogens such as *Hafnia alvei* [23]. Furthermore, the capacity of *M. rotundata* to undergo facultative diapause, a state characterized by enhanced stress tolerance and metabolic reorganization, may be partially mediated by microbiome–host interactions [16,36]. The diapause state involves significant physiological changes, including accumulation of cryoprotectants and alteration of energy metabolism, processes potentially influenced by gut microbial communities [16]. Understanding these microbiome-mediated resilience mechanisms is critical for optimizing commercial management practices, particularly for maintaining bee health during cold storage and ensuring synchronous emergence with crop bloom [37,38].

## 5. Conclusions

This study represents the first systematic comparison of gut microbiome and dietary dynamics among three bee species (*Megachile rotundata*, *Osmia excavata*, and *Apis cerana*) before and after alfalfa pollination. Our findings reveal a dynamic interplay between dietary shifts and microbial community assembly, demonstrating that gut microbiota composition is not solely determined by host phylogeny but is also heavily influenced by environmental factors such as floral resource availability. For instance, while *Apis cerana* exhibited a pronounced reliance on *Cucurbitales* after feeding, its gut microbiota shifted toward carbohydrate-fermenting taxa like *Gilliamella*, aligning with its dietary specialization. In contrast, *Megachile rotundata* maintained a more diverse microbial community, likely reflecting its flexible foraging behavior and ability to adapt to varying dietary conditions. The dominance of *Bacillus* in *Osmia excavata* suggests a potential niche for nitrogen-fixing or pathogen-suppressing symbionts, which could be leveraged to enhance habitat quality in agroecosystems.

By revealing the response patterns of gut microbiota to dietary changes, our study provides a microbial perspective on pollinator health and resilience. The link between microbial diversity and host adaptability underscores the need to preserve not only floral resources but also microbial symbionts as critical components of pollinator habitats. Similarly, understanding the functional roles of key microbial taxa, such as *Gilliamella*’s role in carbohydrate metabolism, could inform the development of probiotic supplements to boost bee nutrition and stress tolerance. Furthermore, our results highlight the potential for microbial symbioses to drive evolutionary divergence among bee species. The specialization of *Apis cerana*’s microbiota on *Cucurbitales*-associated taxa, for instance, may reinforce its foraging preferences over time, leading to niche partitioning and reduced interspecific competition.

In total, this study represents the first integrated analysis of gut microbiome dynamics and dietary composition in three co-occurring pollinator species during active crop pollination. These results provide a microbial foundation for understanding how foraging behavior and sociality interact to shape pollinator health in agricultural ecosystems, offering novel insights for developing species-specific conservation strategies that maintain pollinator diversity while ensuring sustainable crop pollination services.

## Figures and Tables

**Table 1:** Primers used for 16S rRNA gene and chloroplast *rbcL* amplification.

Primer Pair	Sequences	Product Length (bp)	Target Region
515F	5′-GTGCCAGCMGCCGCGGTAA-3′	~290–310	16S rRNA V4
806R	5′-GGACTACHVGGGTWTCTAAT-3′		
*rbcL*2_F	5′-YGATGGACTTACNAGTCTTGATCGTTACAAAGG-3′	~280	*rbcL*
*rbcL*2_R	5′-GNCCATAYTTRTTCAATTTATCTCTTTCAACTTGGATNCC-3′		

## Data Availability

The original contributions presented in this study are included in the article/Supplementary Materials. Further inquiries can be directed to the corresponding author.

## Supplementary Materials

The following supporting information can be downloaded at: https://www.mdpi.com/article/10.3390/insects17060561/s1. Table S1: OTU Abundance and Taxonomy of Gut Microbiota. Table S2: Plant Dietary OTU Abundance from *rbcL* Metabarcoding. Table S3: Alpha Diversity Metrics of Gut Microbial Communities.
